# Cardiovascular response to anticipatory and reactionary postural perturbations in young adults

**DOI:** 10.1113/EP091173

**Published:** 2023-07-17

**Authors:** Patrick Siedlecki, Tanya D. Ivanova, S. Jayne Garland

**Affiliations:** ^1^ School of Kinesiology Western University London Ontario Canada; ^2^ Faculty of Health Sciences Western University London Ontario Canada; ^3^ Department of Physiology & Pharmacology Western University London Ontario Canada

**Keywords:** anxiety, heart rate, postural control, systolic blood pressure

## Abstract

Cardiovascular responses to postural perturbations have been reported, but whether the cardiovascular responses to external perturbations could be executed in anticipation of the perturbation is unknown. The purpose of this study was to determine the effect of anticipated and reactionary perturbations on heart rate (HR) and systolic blood pressure (SBP) responses in healthy young adults. A secondary aim was to determine whether perceived state anxiety scores were correlated with the change in HR response during postural perturbation. Twenty healthy young adults stood on a treadmill and experienced two perturbation conditions (anticipatory vs. reactionary), each with two intensity levels (Step vs. No Step). The HR and SBP were collected continuously. Two‐way repeated‐measures statistical non‐parametric mapping tests were used to compare HR and SBP responses to the perturbations over time (from −3 to +8 s). The results indicated that HR was significantly elevated in the higher intensity perturbations [Step vs. No Step, at 0.56–1.32 s (*P* < 0.0001) and 1.92–3.44 s (*P* < 0.0001) post‐perturbation], while there were no differences in HR between perturbation types (anticipatory vs. reactionary) or in SBP between perturbation types and intensity levels. The perceived state anxiety scores did not differ between perturbation types and intensity levels but were correlated with the change in HR post‐perturbation (*P* = 0.013). We suggest that reflexive mechanisms dominate cardiovascular regulation after anticipatory and reactionary perturbations. The data highlight the cardiovascular mechanism(s) associated with perturbations that should be considered when assessing postural stability in populations with poor balance performance.

## Abstract (highlights)

- **What is the central question of this study?**
  It has been suggested that the cardiovascular responses to a postural perturbation are centrally mediated and reflex mediated. We wanted to know the extent to which the cardiovascular responses to external perturbations could be executed in a feedforward manner, in anticipation of the perturbation.
- **What is the main finding and its importance?**
  We found no anticipatory component driving heart rate and systolic blood pressure responses, suggesting that reflexive mechanisms dominate cardiovascular regulation after a postural perturbation in young adults.

## 1 INTRODUCTION

The relative importance of central versus reflex involvement in cardiovascular responses to postural stress is unknown. Skeletal muscles receive descending neural input from the motor and sympathetic neurons (Barker & Saito, 1981), highlighting the autonomic influence on skeletal muscle function (Blackwood & Katz, 2019; Cairns & Borrani, 2015). Interactions between cardiovascular and postural control systems during standing balance have been suggested (Garg et al., 2014). We have shown previously that external perturbations of the support surface resulted in immediate and transient tachycardia followed by increased systolic blood pressure (SBP; Siedlecki, Shoemaker et al., 2022). The immediate heart rate (HR) response post‐perturbation was suspected to be centrally mediated, followed by a secondary baroreflex response when SBP increased concurrently with the decrease of the HR. The extent to which the cardiovascular responses to external perturbations could be executed in a feedforward manner, in anticipation of the perturbation, is unknown.

Cardiovascular adjustments can be centrally mediated and reflexively mediated (Nobrega et al., 2014). The central regulatory mechanism can be accompanied by an anticipatory component that occurs before the onset of physical work (i.e., cycling in this case) when the timing of the onset is known and the workload is predetermined (Krogh & Lindhard, 1913). A concurrent rise in blood pressure and HR is often associated with the central command for physical exercise and is affected by the type of exercise, intensity of exercise, time after onset and the effectiveness of blood flow in supplying metabolites to working muscles (Mitchell, 2012). The reflexive component, however, can manipulate blood pressure and HR in response to a stimulus (e.g., isometric contraction) and make minor adjustments throughout the task (Grotle et al., 2020). Although central and reflex control mechanisms can work in combination with one another, each system can work in isolation (Dombrowski et al., 2018). Tasks involving an active change in posture (e.g., sit‐to‐stand task) have been described as primarily reactive feedback mechanisms used to regulate blood pressure and HR (Borst et al., 1982; Olufsen et al., 2008). It should be noted that centrally mediated cardiovascular regulation (Siedlecki, Shoemaker et al., 2022) and anticipatory neuromuscular responses (Pollock et al., 2017) during brief postural perturbations have also been reported in postural control. Thus, introducing an anticipatory component to the postural perturbations might reveal a feedforward component of the cardiovascular response to postural perturbations.

State anxiety also has a transient effect on the cardiovascular system at rest (i.e., HR and SBP; Virtanen et al., 2003) and has been observed to be positively correlated with blood pressure during postural control (Carpenter et al., 2006). The anticipatory and reactionary neuromuscular responses to postural perturbations have been well delineated (Bugnariu & Sveistrup, 2006; McIlroy & Maki, 1993; Piscitelli et al., 2017; Walchli et al., 2017). Thus, larger perturbations with unpredictable timing might result in higher anxiety and, therefore, might provoke a larger cardiovascular response to the postural perturbation.

The primary purpose of this study was to determine the effect of anticipated and reactionary perturbations on HR and SBP responses in healthy young adults. A secondary aim was to determine whether perceived state anxiety scores were correlated with the change in HR response during postural perturbation. It was hypothesized that if a centrally mediated response with an anticipatory component were to be driving any cardiovascular changes, anticipated challenges to standing balance would elevate the HR and SBP before postural perturbations, with larger responses occurring in more difficult perturbation tasks. It was also hypothesized that perceived state anxiety scores would be highest in the unpredictable and highest intensity conditions and would be correlated with the change in HR post‐perturbation.

## 2 MATERIALS AND METHODS

### 2.1 Ethical approval

Twenty adults aged between 18 and 35 years, who met the inclusion criteria of having no history of cardiovascular, neurological or respiratory health issues, completed the study. The study was approved by the Western University's Health Sciences’ Research Ethics Board (#110471) and adhered to the principles and practices set out in the *Declaration of Helsinki* (World Medical Association, 2013), except for registration in a database. Before the experimental session, participants refrained from performing strenuous physical activity for 12 h. Additionally, participants avoided consumption of alcoholic and caffeinated beverages and fasted for a minimum of 4 h before visiting the laboratory. Once written consent was obtained, participants completed a health history and medication screening form to ensure that the study inclusion criteria were met.

### 2.2 Experimental protocol

The experimental procedures consisted of administering the community balance and mobility (CB&M) scale and the self evaluation breathing questionnaire, version 2 (SEBQ‐2), followed by a standing balance protocol. The CB&M is a collection of 19 balance tests (e.g., unilateral stance, descending a set of stairs, walking forwards and backwards) that assess ambulatory balance in adults (Howe et al., 2006). Tests are scored by the researcher on a 0‐ to 5‐point interval scale. The highest total score available is 96, which includes one bonus point. Higher scores reflect better balance performance. Participants in a similar age range have been reported to score ∼95 on the CB&M (Siedlecki, Ivanova et al., 2022; Siedlecki, Shoemaker et al., 2022). The SEBQ‐2 is a 25‐item self‐report questionnaire that was used to assess respiratory‐related health issues (Courtney & van Dixhoorn, 2014). Items are ranked on a 4‐point Likert scale, ranging from 0 (never/not true at all) to 3 (very frequently/very true). An overall score of >24 out of 75 indicates the possibility of respiratory‐related health problems (Kiesel et al., 2017).

The standing balance protocol consisted of three conditions: a 5 min quiet standing period and two perturbation types (self‐triggered and computer‐triggered), each with two perturbation intensities. The self‐triggered perturbation (perturbation timing controlled by the participant) was used to enable an anticipatory response, whereas a computer‐triggered perturbation (exact timing of perturbation unknown) was used to stimulate a reactionary response to the loss of balance. There was a total of four perturbation conditions: self‐triggered/anticipatory perturbation causing a Step (STS) and No Step (STN), and computer‐triggered/reactionary perturbation causing a Step (CTS) and No Step (CTN), described in more detail below. All perturbation conditions contained 25 trials occurring every 8–12 s. We have used a similar protocol to measure cardiovascular responses at different perturbation intensities (Siedlecki, Shoemaker et al., 2022).

The Gait in Real‐time Analysis Interactive Lab (GRAIL; Motekforce Link, Amsterdam, The Netherlands) system was used to perform perturbation tests. The GRAIL, consisting of an instrumented split‐belt treadmill in front of a 180° virtual‐reality screen, delivered bilateral, posteriorly directed translations of the treadmill belts (300 ms duration) while participants were standing. Participants wore an upper body safety harness, which was attached to the ceiling; the safety harness did not provide any body weight support. Participants were fitted with a three‐lead Colin Pilot‐9200 Bedside Monitor ECG (Colin Medical, San Antonio, TX, USA) and a finger cuff with brachial Finometer sphygmomanometer (Finapres Medical Systems, Amsterdam, The Netherlands) placed on the right arm to collect continuous cardiovascular measurements. An arm sling worn around the right arm limited the movement of the sphygmomanometer.

Participants were familiarized with the postural perturbations before data collection, as the velocity of the belt movements (perturbation intensity) increased with subsequent perturbations until participants were consistently taking a step to regain balance. The No Step intensity was determined as the highest intensity at which a participant was able to regain balance without taking a step or grabbing the handrails. The Step intensity was set to 50% above the intensity selected for the No Step intensity and caused a reactive stepping response to maintain standing balance. The order of perturbation types was randomized, as was the order of the intensities within the self‐triggered and computer‐triggered conditions. This meant that the Step and No Step intensities of the same perturbation type would always occur consecutively but in a different order across participants.

An application created in the GRAIL software D‐flow (Motekforce Link) was used to trigger treadmill perturbations. A Phidget Analog 4‐output #1002_0B (Phidget, Calgary, AB, Canada) recorded the speed of the treadmill belts. During the self‐triggered perturbation type, a series of 25 perturbations was triggered by a touch sensor that was fixed to the participant's left thigh at a height that did not require a change in posture when touched to trigger the perturbation. The participant was told to wait for verbal instruction (‘Whenever you are ready, touch the button’) and then touch the button 2–3 s (or longer) after notification was received and not to react immediately. After a perturbation was triggered, the button would automatically deactivate until the next trial (8–12 s). The computer‐triggered perturbation type consisted of 25 trials that occurred simultaneously with an auditory cue. To reduce anticipation of the computer‐triggered perturbation by the participant, a perturbation was triggered in only ∼70% of trials, resulting in 17 or 18 perturbations over the 25 trials.

Participants were asked to rate their perceived state of anxiety during quiet stance (QS) and after each performed series of perturbations on a scale from 0 (‘no anxiety’) to 10 (‘the most anxious you have ever felt’). The perceived state anxiety scale was chosen over other anxiety questionnaires and scales owing to quick administration and single‐word responses that would limit arousal during the experimental session.

### 2.3 Blood pressure and heart rate response calculations

The ECG, blood pressure and belt velocity signal traces were sampled at 1000 Hz in Powerlab 8/35 (ADInstruments, Bella Vista, NSW, Australia) to collect beat‐to‐beat HR and SBP data and to identify the onset of perturbation, respectively. The data for each series of perturbations were imported into Spike2 v.8.13 (Cambridge Electronic Design, Milton, UK). The beat‐to‐beat data were down‐sampled to 50 Hz and an 11 s window starting 3 s before the onset of a perturbation was selected for each trial. The rationale for analysing data 3 s before the perturbation was based on the time given to participants to trigger a perturbation in the self‐triggered condition. Trials with artefacts in the HR and SBP traces were excluded. An artefact was defined as a change in HR that was caused by the Powerlab software algorithm incorrectly labelling an ECG waveform or a change in SBP that was caused by pressure from an adjacent finger applying force against the finger cuff. These artefacts produced unrealistic HR (>200 beats/min) and SBP (>300 mmHg) recordings. Individual trials were averaged for each participant within each perturbation type and intensity. Data averaged from the entire 5 min quiet standing period were used for QS.

The HR and SBP data were analysed separately using one‐dimensional statistical parametric mapping (1‐D SPM). In short, continuous data for the dependent variable (HR or SBP) were plotted in the first dimension (from −3 to +8 s). The data were converted into residuals, and an *F*‐statistic was calculated for each individual point (550 time points), creating an *F*‐curve. Random field theory randomly sampled data, calculated the maximum *F*‐value for each permutation across all data points and created a maximum *F*‐statistic distribution for each permutation. The critical *F*‐value (threshold) was determined based on the α‐level (*P* = 0.05) of the maximum *F*‐statistic distribution and signified the maximum *F*‐statistic where the null hypothesis was true. The critical *F*‐value was compared with the *F*‐curve, and any clusters above the critical *F*‐value had a *P*‐value of <0.05, indicating that the null hypothesis must be rejected. Random field theory was used to calculate the specific *P*‐value of each cluster (Adler & Taylor, 2007).

The change in HR was also calculated for all perturbation types and intensities by subtracting the HR at the time of the perturbation onset from the peak HR post‐perturbation.

### 2.4 Statistical analysis

Statistical analyses were performed with SPSS v.25 (IBM SPSS, Armonk, NY, USA), R v.4.2.1 (R Core Team) and MATLAB 2019b (MathWorks, Natick, MA, USA). The HR and SBP responses were compared across the perturbation types and intensities with 1‐D SPM using an open source spm‐1D package (spm1d.org; T. Pataky). The SPM method was selected over traditional scalar extractions because SPM provides a comprehensive analysis of dependent variables as a function of time, without concerns over data reduction, and accounting for multiple comparisons (Pataky et al., 2013). Recently, 1‐D SPM has been validated in human movement sciences for use in biomechanical research to analyse joint torque angles (Pataky et al., 2013; Serrien et al., 2019) over the percentage of stance phase, or EMG signals and muscle forces (Haddara et al., 2020; Robinson et al., 2015) over time. Our study had a greater resolution (*n* = 500) in comparison to those papers (*n* ≈ 100). The 1‐D SPM included all data points throughout our epoch without having to condense the data and choose arbitrary epochs for analysis. A non‐parametric two‐way repeated‐measures ANOVA 1‐D SPM (SnPM) test, for both HR and SBP, was used because the cardiovascular data were not normally distributed. The 1‐D SnPM compared the response of the cardiovascular data as a function of time (from −3 to +8 s) between perturbation type (self‐triggered and computer‐triggered) and intensity (Step and No‐Step).

A one‐way repeated‐measures ANOVA was performed to evaluate the difference in perceived state anxiety scores across conditions (QS and perturbations). If a significant difference was found, a Bonferroni post‐hoc analysis was used to identify where the statistical difference occurred. A second analysis to determine the relationship between state anxiety scores and the change in HR post‐perturbation was performed with a repeated‐measures correlation using the ‘rmcorr’ R package. The repeated‐measures correlation satisfies the assumption of independent observations and has greater statistical power than other simple correlation tests (Bakdash & Marusich, 2017).

The level of significance was set to *P* < 0.05 for all statistical tests mentioned above. Data are presented as the mean (SD) unless otherwise stated.

## 3 RESULTS

The characteristics for all 20 participants can be found in Table 1. Data from two participants were excluded from the analysis owing to incomplete HR data as a result of technical issues during data collection. During QS, the mean HR across participants was 84 beats/min (SD 12 beats/min), and SBP was 118 mmHg (SD 13 mmHg).

**TABLE 1:** Participant characteristics.

Age (years)	Sex^a^ (M/F)	Height (cm)	Weight (kg)	SEBQ scores (/75)	CM&M score (/96)
24 (3.7)	10/10	172.5 (8.8)	70.9 (14.8)	4.8 (5.4)	95.6 (0.6)

*Note*: Data are presented as the mean (SD).

Abbreviations: CB&M, community balance and mobility scale; F, female; M, male; SEBQ, self‐evaluation breathing questionnaire.

^a^ Number of participants.

### 3.1 Cardiovascular response to postural perturbations

**FIGURE 1:**
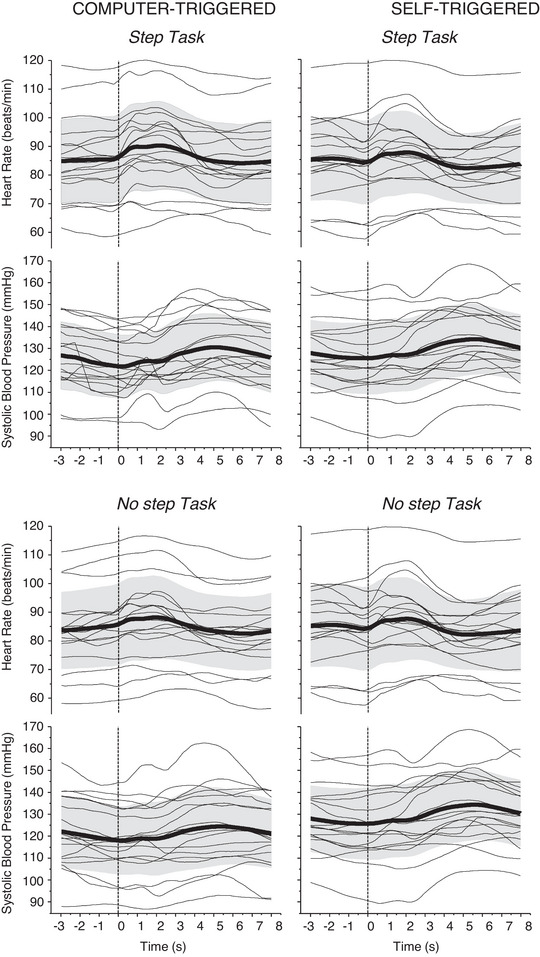
Heart rate (HR) and systolic blood pressure (SBP) responses for computer‐triggered (left panels) and self‐triggered (right panels) perturbation conditions with Step (top panels) and No Step (bottom panels) perturbation intensities (*n* = 18). The group means and SDs are presented as bold black lines and grey shaded areas, respectively. The HR and SBP (average across trials) of individual participants are represented with thin lines. The dashed vertical line indicates the onset of the perturbation.

The mean HR and SBP during the four postural perturbation tasks for each individual are illustrated in Figure 1. It is apparent from Figure 1 that cardiovascular responses to the perturbation were occurring after the perturbation and were not starting before the perturbation in anticipation of the self‐triggered perturbation type.

**FIGURE 2:**
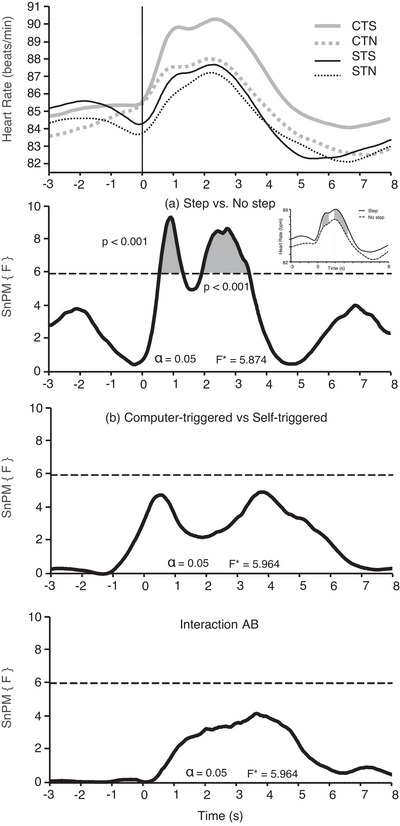
Statistical non‐parametric mapping (SnPM) analysis for heart rate (HR; *n* = 18). In the top panel are presented the average values of HR in computer‐triggered Step (CTS; grey continuous line), computer‐triggered No Step (CTN; grey dotted line), self‐triggered Step (STS; black solid line) and self‐triggered No Step (STN; black dotted line) tasks, with the onset of the perturbation indicated by a vertical line at time 0. The three panels below contain the plots of the SnPM *F*‐statistic (continuous black lines) for the effect of perturbation intensity [(a) Step vs. No step], for the effect of perturbation type [(b) computer‐triggered vs. self‐triggered] and for the interaction between perturbation type and intensity (Interaction AB). Dashed horizontal lines in each panel represent the critical threshold for the non‐parametric SPM tests. An inset in panel ‘(a) Step versus No step’ shows the HR averaged across perturbation types: Step (continuous line) and No Step (dotted line), with the periods of significant differences between tasks highlighted in grey.

The average HR response in the self‐triggered and computer‐triggered perturbation types at two intensities is illustrated in Figure 2. The SnPM identified two periods (clusters) in which there was a significant effect of intensity on HR (F* = 5.874). Heart rate rose earlier in the Step Perturbation (identified between 0.56 and 1.32 s; *P* < 0.0001) and remained elevated for longer in the falling phase (identified between 1.92 and 3.44 s; *P* < 0.0001) compared with the No Step Perturbation. The inset in Figure 2 shows this effect when the self‐ and computer‐triggered conditions were combined. There was no statistically significant interaction effect between perturbation type and intensity (F* = 5.964) or a main effect of perturbation type (F* = 5.964) for HR.

**FIGURE 3:**
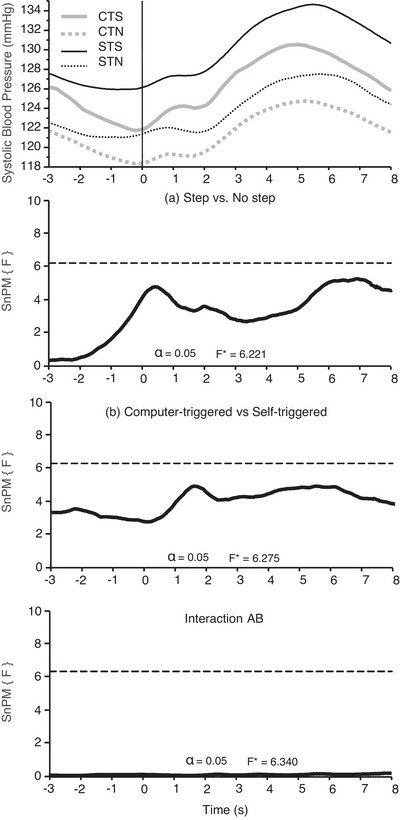
Statistical non‐parametric mapping (SnPM) analysis for systolic blood pressure (SBP; *n* = 18). In the top panel are presented the average SBP in computer‐triggered Step (CTS; grey continuous line), computer‐triggered No Step (CTN; grey dotted line), self‐triggered Step (STS; black continuous line) and self‐triggered No Step (STN; black dotted line) tasks, with the onset of the perturbation indicated by a vertical line at time 0. The three panels below contain the plots of the SnPM *F*‐statistic (continuous black lines) for the effect of perturbation intensity [(a) Step vs. No step], for the effect of perturbation type [(b) computer‐triggered vs. self‐triggered] and for the interaction between perturbation type and intensity (Interaction AB). Dashed horizontal lines in each panel represent the critical threshold for the non‐parametric SPM tests.

No statistically significant interaction effect between perturbation type and intensity (F* = 6.34) or main effect of perturbation type (F* = 6.221) or main effect of intensity (F* = 6.275) was found for SBP. The average SBP responses during the perturbations are shown in Figure 3.

### 3.2 Perturbation effect on perceived state anxiety

**FIGURE 4:**
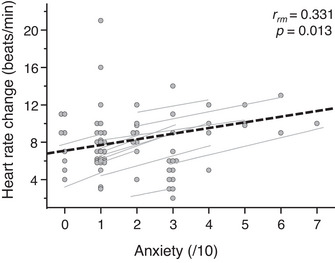
The repeated‐measures correlation between the change in heart rate (HR) and state anxiety scores following postural perturbations (bold dashed black line; *n* = 18). The data for each participant, consisting of the individual peak change in HR (averaged across trials) versus anxiety score after a perturbation task (filled circle) and the line of best fit (thin continuous line), are plotted. The overall line of best fit and the correlation coefficient were calculated from the above‐mentioned lines of best fit for all participants.

There was a difference in perceived state anxiety scores between QS [1.1 (SD 1.1)], STS [1.7 (SD 1.3)], STN [1.5 (SD 1.0)], CTS [2.7 (SD 1.8)] and CTN [1.8 (SD 1.4)] perturbations [*F*_4,68_ = 7.485, *P* = 0.0003, 1 − β = 0.979]. Perceived anxiety was rated higher in CTS compared with QS (*P* = 0.002), but there was no difference between QS and STN (*P* = 1.00), STS (*P* = 0.962) or CTN (*P* = 0.187). There were also no statistical differences in anxiety scores between STS and STN (*P* = 1.00), between CTS and CTN (*P* = 0.142), between STS and CTS (*P* = 0.096) and between STN and CTN (*P* = 1.00) perturbations. Although the only group difference in state anxiety data was between CTS and QS, a repeated‐measures correlation analysis found that there was a relationship between the state anxiety scores and the change in HR post‐perturbation (*r*_rm_= 0.331, *P* = 0.013; Figure 4), indicating that after a perturbation, within individuals the higher state anxiety scores were moderately correlated with greater changes in HR.

## 4 DISCUSSION

The main outcomes of the study are as follows: (1) the HR response scaled with the intensity of the perturbation; (2) knowing the timing of the perturbation had no effect on HR and SBP before and after the postural perturbation; and (3) the perceived state anxiety scores did not differ by perturbation condition but were correlated with the change in HR post‐perturbation. These findings support the null hypotheses, in part.

### 4.1 Cardiovascular response to postural perturbations

No anticipatory response before the perturbation was found for HR and SBP when the timing of the perturbation was known. The similarities between pre‐perturbation and QS HR and SBP also suggests a lack of an anticipatory response to perturbations. Additionally, a similar HR and SBP response occurred post‐perturbation regardless of whether the timing of the perturbation was known. Postural perturbations, regardless of intensity and knowing the timing of the perturbation or not, elicited immediate tachycardia followed by elevated SBP, with HR recovering before the SBP recovery. The SBP response was not intensity dependent, whereas HR was elevated in the Step compared with No Step task during the initial rise in HR (from 0.56 to 1.32 s) and during the recovery (from 1.92 to 3.44 s). Although the exact timing of these time periods might not be essential, their location in the HR response post‐perturbation is important. In the first time period (cluster), immediately after the perturbation, the significant difference between the tasks indicated that HR rose more quickly and reached a higher peak earlier in the Step compared with No Step tasks, whereas in the second time period, HR remained elevated for longer in the Step compared with No Step tasks. The HR and SBP responses post‐perturbation were in agreement with previous findings when the exact timing of the perturbation was unknown (Siedlecki, Shoemaker et al., 2022).

The similar findings between self‐ and computer‐triggered perturbations could be attributable, in part, to the emotional response to the perturbations. Anticipatory HR and blood pressure changes can be affected by the effortful nature of a task (Gandevia et al., 1993; Morgan et al., 1973). Volitional limb movements alone are not enough to evoke an anticipatory cardiovascular response, because a movement must also be accompanied by emotional content (Smith et al., 2000). The self‐triggered postural perturbations were not accompanied by lower state anxiety scores than computer‐triggered perturbations. It should be noted that perceived anxiety scores only increased during computer‐triggered Step perturbations despite HR response scaling with intensity in both perturbation conditions.

Although elevated state anxiety enhances the physiological arousal of the sympathetic nervous system, consequently triggering tachycardia during stressful events (Dimitriev et al., 2016), it would appear that the participants did not consider any of these perturbations to be particularly anxiety provoking, given that most participants rated anxiety <4 out of 10 during perturbation tasks. The difference in anxiety scores between the intervention and placebo conditions can vary by ∼10 units when using the state‐trait anxiety inventory (Bae et al., 2019), further suggesting that postural perturbations in the present study might not have been perceived as being challenging. Although the timing of the perturbation was unknown in the computer‐triggered condition, participants still knew that they would be perturbed. However, the CTS perturbations might have been perceived as more challenging than the other perturbation tasks because the timing of the perturbation was unknown and required a step to regain balance. A commonly used inventory to rate state anxiety is the state‐trait anxiety inventory, in which state anxiety scores range between 40 and 80 units (Spielberger et al., 1983). Following psychological stress, salivary cortisone, a biomarker for stress, is moderately correlated with state‐trait anxiety inventory scores and strongly correlated with HR response (Bae et al., 2019). Our findings support this notion, because state anxiety scores in the present study were moderately correlated with the change in HR post‐perturbation. This might suggest that anxiety influenced the amplitude of the initial cardiac response, because more anxiety‐provoking perturbations induced greater increases in HR.

In our previous study, the immediate HR and blood pressure responses to postural perturbations were thought to be centrally mediated, with a secondary baroreflex response to aid recovery (Siedlecki, Shoemaker et al., 2022). It is possible that postural perturbations do not illicit an anticipatory response, because centrally mediated haemodynamics occur only after a change in posture. Patel et al. (2018) examined HR responses during anticipated passive head‐up tilts in a feline model. Their protocol consisted of a light flash 30 s before a passive 60° head‐up tilt with randomly inserted 20° head‐up tilts. They found no anticipatory HR response 5 s before the onset of movement, whereas immediately after the onset of the movement the HR increased. We found a similar HR response in the post‐perturbation time frame in the present study.

The lack of a difference between anticipatory and reactionary perturbations on the initial cardiovascular response might suggest that a non‐cardiovascular‐mediated mechanism was responsible, in part, for the rapid increase in HR post‐perturbation. Although participants were instructed to limit head movement, the horizontal acceleration of the body immediately after the perturbation would have activated the vestibular system, because the otolith organs sense linear acceleration. The cardiovascular system and vestibular apparatus share neural pathways within the rostral ventrolateral medulla in cats (Gagliuso et al., 2019; Yates et al., 1993, 1991), and the effect of vestibular inputs on haemodynamics has been reported in humans (Bent et al., 2006; Yates et al., 1999). When horizontally accelerated, a rapid and immediate increase in HR and blood pressure occurs (Goldberg & Fernandez, 1984) that is independent of psychological arousal elicited by the task (Yates et al., 1999). Thus, activation of the vestibular system might explain, in part, why HR increased rapidly post‐perturbation although there was no difference between perceived state anxiety scores between perturbation tasks.

A similar experimental protocol examining the postural response to anticipatory perturbations showed that the neuromuscular responses were anticipatory in a self‐triggered perturbation (Pollock et al., 2017). Given that the self‐triggered perturbations in the present study were similar to the perturbations in the study by Pollock et al. (2017), it is likely that the sensorimotor system anticipated the postural perturbations, whereas the cardiovascular system did not.

### 4.2 Limitations

A limitation of the present study was the inability to distinguish between whether young adults did not perceive the perturbations as being anxiety provoking or whether the perceived state anxiety scale was not sensitive enough to discriminate between the perturbation types and intensities.

### 4.3 Future directions

Our results indicated that anticipatory cardiovascular responses to postural perturbations were absent when the timing of the perturbation was known. Furthermore, knowledge of the exact timing of the perturbation did not affect the HR and SBP responses, suggesting the importance of reflex‐mediated haemodynamics following a perturbation. In addition, the computer triggered step‐evoking perturbation might have been perceived as the most challenging task, as noted by higher perceived state anxiety scores. Higher anxiety scores were related to greater changes in HR during the initial cardiac response. Future focus should be placed on the effects of postural perturbations on the cardiovascular response in older adults, because this population is known to have age‐associated cardiovascular (Karavidas et al., 2010; Paneni et al., 2017) and balance impairments (Paillard, 2017; Roman‐Liu, 2018). Thus, the generalization of our findings is limited to young adults.

## 5 CONCLUSION

Comparable haemodynamics between self‐ and computer‐triggered postural perturbation types suggest that similar mechanisms are responsible for cardiovascular regulation immediately after a postural perturbation. Any effects of anticipation on cardiovascular responses before the perturbation are believed to be minimal. It is also likely that individual anxiety levels influenced the change in HR during the initial cardiac response.

## AUTHOR CONTRIBUTIONS

Patrick Siedlecki conceived, designed and conducted the experiment. Patrick Siedlecki and Tanya D. Ivanova analysed the data. Patrick Siedlecki, Tanya D. Ivanova and S. Jayne Garland interpreted results of the experiment. Patrick Siedlecki drafted the manuscript. S. Jayne Garland supervised the study. All authors revised, read and approved the final version of the manuscript and agree to be accountable for all aspects of the work in ensuring that questions related to the accuracy or integrity of any part of the work are appropriately investigated and resolved. All persons designated as authors qualify for authorship, and all those who qualify for authorship are listed.

## CONFLICT OF INTEREST

None declared.

## FUNDING INFORMATION

This research project did not receive funding.

## Supporting information


Statistical Summary Document

## Data Availability

The data that support the findings of this study are available on request from the corresponding author.
